# *Nicotinic Acetylcholine Receptor* Variants Are Related to Smoking Habits, but Not Directly to COPD

**DOI:** 10.1371/journal.pone.0033386

**Published:** 2012-03-15

**Authors:** Simona E. Budulac, Judith M. Vonk, Dirkje S. Postma, Mateusz Siedlinski, Wim Timens, Marike H. Boezen

**Affiliations:** 1 Department of Epidemiology, University Medical Center Groningen, University of Groningen, Groningen, The Netherlands; 2 Department of Pulmonology, University Medical Center Groningen, University of Groningen, Groningen, The Netherlands; 3 Department of Pathology, University Medical Center Groningen, University of Groningen, Groningen, The Netherlands; 4 Groningen Research Institute for Asthma and COPD (GRIAC), University Medical Center Groningen, University of Groningen, Groningen, The Netherlands; University of Illinois at Chicago, United States of America

## Abstract

Genome-wide association studies identified single nucleotide polymorphisms (SNPs) in the *nicotinic acetylcholine receptors (nAChRs)* cluster as a risk factor for nicotine dependency and COPD. We investigated whether SNPs in the *nAChR* cluster are associated with smoking habits and lung function decline, and if these potential associations are independent of each other. The SNPs rs569207, rs1051730 and rs8034191 in the *nAChR* cluster were analyzed in the Vlagtwedde-Vlaardingen cohort (n = 1,390) that was followed for 25 years. We used GEE and LME models to analyze the associations of the SNPs with quitting or restarting smoking and with the annual FEV_1_ decline respectively. Individuals homozygote (CC) for rs569207 were more likely to quit smoking (OR (95%CI) = 1.58 (1.05–2.38)) compared to wild-type (TT) individuals. Individuals homozygote (TT) for rs1051730 were less likely to quit smoking (0.64 (0.42; 0.97)) compared to wild-type (CC) individuals. None of the SNPs was significantly associated with the annual FEV_1_ decline in smokers and ex-smokers. We show that SNPs in the *nAChR* region are associated with smoking habits such as quitting smoking, but have no significant effect on the annual FEV_1_ decline in smokers and ex-smokers, suggesting a potential role of these SNPs in COPD development via smoking habits rather than via direct effects on lung function.

## Introduction

Cigarette smoking is a major concern influencing public health worldwide. It is the main risk factor for Chronic Obstructive Pulmonary Disease (COPD), but the risk to develop airway obstruction varies between smokers due to differences in genetic susceptibility.

Over time, studies in animal models [1], [2], candidate gene or family and twin studies [3], [4] have tackled the underlying mechanisms and genetic background influencing nicotine addiction and smoking habits [5]. Recent reviews highlighted the important role of genetic factors in the inter-individual variation to initiate, maintain or to quit smoking and their effects on lung function [6], [7].

The genetic contribution to the variation in smoking behaviour recognizes two classes of genes: genes influencing the response to nicotine, like nicotinic acetylcholine receptors (*nAChR*) and nicotine metabolism (*CYP2D6*), and genes predisposing to addictive behaviour due to their effects on key neurotransmitter pathways like dopamine (*DRD1*) and serotonin (*TPH*) [8].

*Nicotinic acetylcholine receptor (nAChR)* is a controversial gene with respect to its association with COPD and smoking addition. Single nucleotide polymorphisms (SNPs) rs1051730 and rs8034191 in the *nAChR* cluster have been identified in cross-sectional genome-wide association (GWA) studies as a risk for COPD [9]. Furthermore, studies showed cross-sectional associations of the SNPs in the *nAChR* cluster with nicotine dependency based on the reported number of cigarettes per day [10] or the Fagerstrom Test of Nicotine Dependence (FTND), a test reliably predicting smoking cessation and correlating with biochemical measurements of nicotine dependency [11]. Other studies have found a cross-sectional association of the same variants with the level of lung function and COPD [12].

Since smoking is a risk factor for COPD itself, it is not clear from these cross-sectional studies whether these SNPs in the *nAChR* cluster are directly and independently a risk for COPD development or whether they are associated with COPD through their association with nicotine dependency and smoking habits [10], [11], [13].

We had the unique opportunity to study longitudinally the association of the *nAChR* variants with changes in smoking habits and lung function in the population-based Vlagtwedde-Vlaardingen cohort.

## Results

The clinical characteristics of the Vlagtwedde-Vlaardingen cohort are presented in table 1.

**Table 1:** Clinical characteristics of the Vlagtwedde-Vlaardingen cohort at the last survey.

	Vlagtwedde-Vlaardingen cohort (n = 1,390)
Duration of follow-up (years)	21 (16–22)
Median nr. of visits	7 (5–8)
Males, number (%)	714 (51.4)
Age (years)*	52 (43–60)
Height (cm)	170.3 (9.3)
Never smokers, number (%)	445 (32.0)
Ex-smokers, number (%)	456 (32.8)
Current smokers, number (%)	489 (35.2)
Packyears *	8.9 (0–24)
FEV_1_ (L)	2.8 (0.7)
VC (L)	3.8 (0.9)
FEV_1_/VC (%)	73.8 (8.9)
FEV_1_ (% predicted)	92.6 (15.4)
FEV_1_ change (ml/year)	−20.8 (22.9)
No COPD, number (%)	993 (73.8)
COPD, GOLD stage I, number (%) #	185 (13.8)
COPD, GOLD stage II, number (%) #	146 (10.9)
COPD, GOLD stage III, number (%) #	21 (1.6)

Data are presented as mean (standard deviation) or * median (range);

FEV_1_ = forced expiratory volume in one second; VC = vital capacity; COPD = Chronic Obstructive Pulmonary Disease; GOLD = Global Initiative for Chronic Obstructive Lung Disease; GOLD stage I = mild COPD; GOLD stage II = moderate COPD; GOLD stage III = severe COPD; # According to ATS/ERS standards [35].

The 3 SNPs in the *nAChR* cluster rs1051730, rs8034191 and rs569207 were all in Hardy Weinberg Equilibrium (p>0.05) and their prevalence was relatively high (9% to 57%; Table S1).

### 1. SNPs in the *nAChR* cluster and changes in smoking habits in smokers and ex-smokers

**Figure 1:**
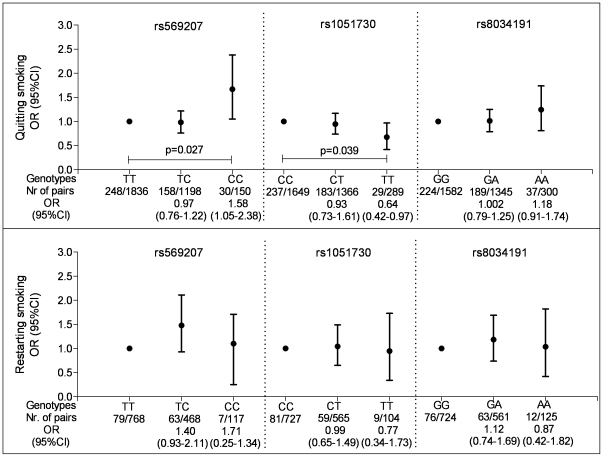
SNPs in the *nAChR* cluster and OR (95%CI) for quitting smoking in subjects who smoke (upper graph), and OR (95%CI) for restarting to smoke in ex-smokers (lower graph). Nr. of pairs = number of paired observations in which the subject stopped respectively restarted smoking/ total number of paired observations included in the analysis; Circles represent the odd ratio (OR) and vertical bars represent 95% confidence interval (CI); Wild type was set to one as the reference category; Different total number of paired observations for the SNP genotypes are due to missing data on genotype or smoking habits. The analyses are adjusted for gender and the time between 2 successive surveys. rs569207 TT = wild-type, TC = heterozygote, CC = homozygote variant rs1051730 CC = wild-type CT = heterozygote TT = homozygote variant rs8034191 GG = wild-type, GA = heterozygote, AA = homozygote variant.

In smokers, individuals homozygote variant (CC) for rs569207 were more likely to quit smoking (OR (odds ratio) (95%CI (confidence interval)) = 1.58 (1.05–2.38)) compared to wild type (TT) individuals (Figure 1, upper graph). Individuals homozygote variant (TT) for rs1051730 were more likely to continue smoking (OR (95%CI) = 0.64 (0.42–0.97)) compared to wild type (CC) individuals (Figure 1, upper graph). The rs8034191 SNP was not associated with quitting smoking. None of the SNPs were associated with restarting smoking (Figure 1, lower graph). Heterozygotes showed no significant associations compared to wild-types. Detailed data on these associations are presented in the supplementary material (Table S2).

### 2. SNPs in the *nAChR* cluster and annual FEV_1_ decline in smokers and ex-smokers

The numbers of available FEV_1_-pairs in smokers and ex-smokers are presented in the supplementary material (Table S3).

None of the SNPs was significantly associated with the annual FEV_1_ decline in smokers or ex-smokers, with or without correction for quitting and restarting smoking, respectively (Table 2). Also, there was no association between the SNPs and the annual FEV_1_ decline in never smokers (Table 2). Additionally we investigated the associations of smoking status with the decline in annual FEV_1_ using an LME model, independent of the genotypes. There was a significant difference in annual FEV_1_ decline between smokers and never smokers and between smokers and ex-smokers independent of the genotypes. Detailed results are presented in the data supplement Table S4.

**Table 2:** SNPs in the *nAChR* cluster and annual FEV_1_ decline (ml/year) in smokers, ex-smokers and never smokers.

SNPs		annual FEV_1_ decline (smokers)^1^			annual FEV_1_ decline (ex-smokers)^2^			annual FEV_1_ decline (never smokers)^3^		
		B	95%CI	p	B	95%CI	p	B	95%CI	p
**rs569207**	a	0.3	−7.6–8.3	0.939	4.1	−6.4–14.5	0.451	2.6	−5.5–10.6	0.531
	b	13.1	−5.7–31.9	0.172	0.9	−15.9–17.8	0.916	2.9	−13.7–19.7	0.727
**rs1051730**	a	0.1	−7.8–8.1	0.973	−2.4	−12.3 – 7.6	0.643	−5.3	−13.1 - 2.6	0.189
	b	5.4	−8.2–18.9	0.437	3.9	−14.3–22.2	0.674	−0.7	−15.1 - 13.6	0.925
**rs8034191**	a	−1.1	−9.2 – 7.1	0.919	−0.3	−10.2 – 9.8	0.964	−3.6	−11.4 - 4.3	0.370
	b	3.9	−9.6–17.4	0.405	5.4	−11.5–22.3	0.531	−4.8	−18.6 - 9.1	0.499

B = regression coefficient from the linear mixed-effect model, adjusted for 1 = quitting smoking, 2 = restarting smoking, 3 = never smoker, gender, height and age at the first of two successive surveys and time between two successive surveys. “Smokers” refer to those paired observations in which the subject was a smoker at the first of two successive surveys and quitted smoking or continued smoking at the nearest follow-up survey. “Ex-smokers” refer to those paired observations in which the subject was an ex-smoker at the first of two successive surveys and continued being an ex-smoker or restarted smoking at the nearest follow-up survey. “Never smoker” refer to those paired observations in which the subject was a never smoker at the first of two successive surveys and continued being a never smoker at the nearest follow-up survey. a = heterozygotes vs. wild-type; b = homozygote variant vs. wild-type.

### 3. Smoking habits and annual FEV_1_ decline in smokers and ex-smokers

Subjects who quitted smoking had (B (regression coefficient) (95%CI) = 20.3 ml (9.49–31.15)) significantly less FEV_1_ decline per year than subjects who continued smoking. Ex-smokers who restarted smoking had a faster FEV_1_ decline (B (95%CI) = −14.7 ml (−31.5 - 2.1)) per year than ex-smokers who did not restart smoking, although this did not reach significance.

### 4. SNPs in the *nAChR* cluster and cigarettes smoked per day, packyears, and COPD at the last survey

In current smokers none of the SNPs was significantly associated with the number of cigarettes smoked per day at the last survey (Table 3).

**Table 3:** SNPs in the *nAChR* cluster with cigarettes smoked per day in current smokers, and packyears in ever smokers at the last survey.

SNP		cigarettes/dayin current smokers*			packyearsin ever smokers**		
		B	95%CI	p-value	B	95%CI	p-value
**rs569207**	a	−1.5	−3.3 - 0.4	0.111	−1.5	−4.1 - 1.0	0.238
	b	−2.8	−7.6 - 2.0	0.252	−5.9	−11.5 - −0.3	0.039
**rs1051730**	a	1.1	−0.6–2.9	0.207	0.1	−2.4–2.6	0.943
	b	−1.5	−4.6 - 1.6	0.338	0.3	−4.1–4.7	0.901
**rs8034191**	a	0.2	−1.6–2.0	0.819	−0.7	−3. - 1.9	0.600
	b	−1.2	−4.3 - 1.9	0.469	−0.6	−4.8 - 3.7	0.800

B = regression coefficient derived from linear regression model, adjusted for age and gender;

* only current smokers at the last survey;

** ever smokers are current smokers and ex-smokers together.

a = heterozygote vs. wild-type.

b = homozygote variant vs. wild-type.

In current and ex-smokers, only individuals homozygote variant (CC) for rs569207 had a lower number of packyears (B (95%CI) = −5.9 (−11.5 - −0.3)) at the last survey as compared to wild-type (TT) individuals (Table 3).

## Discussion

The nAChRs are highly expressed in the nervous system and their binding to nicotine activates physiological and pharmacological responses to tobacco smoking [11]. Variants in the *nAChR* cluster have been associated with nicotine dependency and smoking status [10], [11], [14]. So far, studies showed cross-sectional associations of the *nAChR* variants with nicotine dependency and the level of lung function and COPD [10]–[12], [15]–[18]. Since smoking is a risk factor for COPD itself, cross-sectional studies can not elucidate whether the effect of the *nAChR* variants determine COPD development directly or indirectly via smoking addiction.

**Figure 2:**
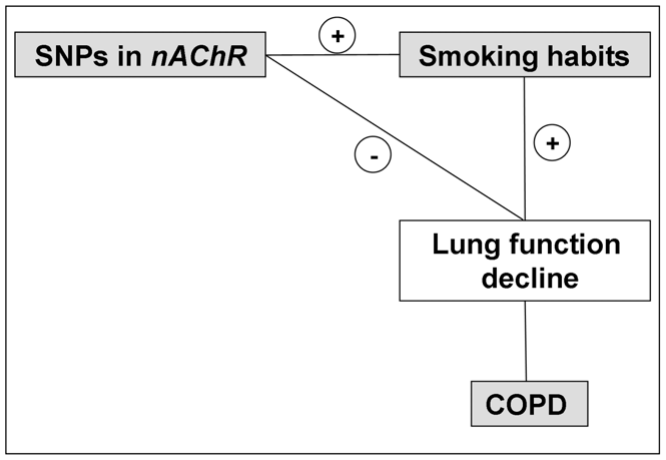
Summary of the observed associations in the current study. SNPs = single nucleotide polymorphisms; COPD = Chronic Obstructive Pulmonary Disease; *nAChR* = *nicotinic acetylcholine receptor*; + = association; − = no association.

Our longitudinal study shows that rs1051730 and rs569207 in the *nAChR* are associated with an increased and decreased ability to quit smoking, respectively, but the SNPs are not associated with the annual FEV_1_ decline. This suggests that these SNPs may be involved in COPD development via smoking habits rather their effect on (accelerated) lung function decline (Figure 2).

First, we observed in smokers that individuals homozygote variant (CC) for rs569207 were more likely to quit smoking compared to wild type (TT) individuals. This is compatible with a previous study showing that the haplotype for rs569207 confers protective effects on nicotine addiction compared with haplotypes for other SNPs within the same Linkage Disequilibrium (LD) block [13]. We additionally found that individuals homozygote variant (TT) for rs1051730 were less likely to quit smoking compared to wild type (CC) individuals, a finding that is in line with a previous study [10]. Although r^2^ between rs569207 and rs1051730 was only 0.11 in our study population (Figure S1), we observed that individuals having the homozygote variant for rs569207 are also the ones who have the wild type for rs1051730 (and thus are less likely to quit smoking), strengthening our findings of a protective effect of the variant in rs569207 for smoking habits within the *nAChR* cluster. Rs1051730 and rs8034191 were highly correlated (r^2^ = 0.91), yet rs8034191 was not associated with quitting smoking among smokers, suggesting that other factors at the subject's level might explain the significant association of rs1051730 with the inability to quit smoking. Moreover, although rs8034191 does not encode an actual receptor protein, we have selected this SNP for analyses with smoking habits since it has been identified in a previous GWA study as a risk for COPD [9].

Second, none of the *nAChR* SNPs was significantly associated with annual FEV_1_ decline in smokers or ex-smokers and neither with annual FEV_1_ decline in never smokers. Additionally, none of the SNPs was significantly associated with the level of FEV_1_ among smokers, ex-smokers and never smokers (data not shown). Pillai et al performed a GWA study in four independent populations and demonstrated that the 15q/25 region is associated with COPD defined by airway obstruction [9]. The authors suggested that the rs1051730 (*CHRNA3*) or rs8034191 (*LOC123688*) are causal variants for COPD [19]. In line with this, rs1051730 and rs8034191 were found to be associated with lower levels of FEV_1_ in the British 1958 birth cohort. However, the latter two studies looked into cross-sectional level of FEV_1_, and thus they cannot disentangle whether the SNPs act via nicotine dependency, or have independent effects. Our longitudinal data strongly suggest that these SNPs are not associated with lung function decline since we found no effect of the SNPs on FEV_1_ decline, and additionally that this lack of effect was independent of smoking.

Third, we observed that smokers who quit smoking had less annual FEV_1_ decline compared with subjects who continued smoking. Our findings are consistent with the results from previous studies demonstrating in a smoking cessation program that quitters had less FEV_1_ decline compared with persistent smokers with COPD [20].

Results of two independent cohorts showed that rs1051730 is additionally associated with emphysema, a feature of COPD, but not with nicotine addiction when defined as the number of packyears smoked [12]. We also found that neither rs1051730 nor rs8034191 were associated with the number of packyears smoked at the last survey suggesting that the number of packyears does not necessarily reflect addiction. Interestingly, in current and ex-smokers homozygote variant (CC) for rs569207 had a lower number of packyears at the last survey than wild type (TT) individuals. This may reflect that carriers of this variant are able to quit smoking more easily, highlighting the consistency of our results.

Our longitudinal results draw the attention to rs569207 in the CHRNA5 gene. This SNP showed a protective effect in relation to smoking habits in the general population, without an effect on annual FEV_1_ decline. This clearly suggests that rs569207 has a protective effect on COPD development via smoking habits exclusively and not via lung function decline (Figure 2). This increased likelihood of quitting smoking was previously seen in individuals carrying *CYP2A6* variants [21]. *CYP2A6*, the gene that influences the response to nicotine besides *nAChRs*, is responsible for the metabolic inactivation of nicotine to cotinine [22]. It is tempting to speculate that as for *CYP2A6* variants, individuals carrying rs569207 do not build up a tolerance to nicotine which is thought to play a critical role in the development and maintenance of nicotine dependency. However, this seems to be unlikely since rs569207 is located in intron 1 and might not have effects other than the regulation of the gene/protein expression. So, from the current study we can not draw firm conclusions on the biological effects of the SNPs in *nAChR* cluster and future functional studies should focus on this in detail. Moreover, pre-clinical studies have demonstrated that bupropion is a non-competitive antagonist at the *nAChRs* subunits [23]–[25] and thus it is plausible that genetic variation in *CHRNA5* influences bupropion's efficacy for treatment of tobacco dependence [25]. It has been shown that rs871058, an intronic SNP in *CHRNA5*, appears to have pharmacogenetic relevance [25]. This intronic SNP rs871058 is highly correlated with rs16969968 in *CHRNA5*. The latest emerged as the strongest risk variant in a nicotine dependence association study of over 3,000 SNPs in over 300 candidate genes [14], [25]. Therefore, it might be that the intronic SNP, rs569207, from the current study is in strong linkage disequilibrium with other SNPs that are able to alter the subunit properties or even have a pharmacological effect, since there is a high degree of linkage disequilibrium across *CHRNA5* [25]. Based on our data we can not state that our observations are the results of monogenic effects. Furthermore, pharmacogenetic variation can significantly alter susceptibility to, and response to treatment for, drug dependence [26]. In a recent study it has been hypothesized that slower nicotine metabolism, as measured by the nicotine metabolite ratio, would decrease the influence of genotypes for rs16969968, rs1051730 in *CHRNA5* and rs578776 in *CHRNA3* [27]. The authors found, in a sample of 1030 treatment seekers, significant independent and additive associations of nicotine metabolite ratio, rs16969968, and rs1051730 with cigarettes per day, but the interactions of the nicotine metabolite ratio with genotype on cigarettes per day were not significant [27]. A recent randomized clinical trial of nicotine replacement therapy showed that fast nicotine metabolizers are less likely to succeed at quitting smoking, as compared to slow nicotine metabolizers, when offered free nicotine patches [28].

This is the first longitudinal study suggesting that SNPs in the *nAChR* cluster potentially have a causal role in COPD via smoking habits. We hypothesize therefore that these variants are related to the onset of COPD via their association with smoking habits, and they are not independently related to COPD development.

## Methods

### Ethics Statement

The study protocol was approved by the local university medical hospital ethics committee, University Medical Center Groningen, University of Groningen, The Netherlands and all patients gave their written informed consent. In 1984, the Committee on Human Subjects in Research of the University of Groningen reviewed the study and affirmed the safety of the protocol and study design (http://www.ccmo-online.nl/main.asp?pid=14&sid=16&ssid=33&inid=16).

### Study population

The Vlagtwedde-Vlaardingen cohort (n = 1390) has been previously described in detail [29], [30]. The cohort was prospectively followed for 25 years with lung function measurements every 3 years using a water-sealed spirometer (Lode Intruments, the Netherlands). Lung function was determined by measurement of the Forced Expiratory Volume in 1 second (FEV_1_). The median number of surveys during the follow-up was 7. Current, ex-smokers and never smokers definition is based on validated questionnaires. A current smoker has been defined as having smoked in the previous 12 months and an ex-smoker as not having smoked in the previous 12 months.

### Selection and genotyping of the SNPs in the *nAChR* cluster

We selected three SNPs rs1051730, rs8034191 and rs569207 in the *nAChR* cluster based on previous findings [9], [11]–[13], [31], [32]. The SNPs belong to the linkage-disequilibrium block containing *nAChR* genes, but do not represent the overall CHRNA5-A3-B4 group. Rs1051730 is a synonymous mutation and rs569207 an intronic SNP belonging to the *nAChR* subunit genes, *CHRNA3* and *CHRNA5* respectively, and the third SNP, rs8034191, is located near *nAChR* subunit genes on 15q25 on *LOC123688*. Genotyping was performed by K-Bioscience (UK) using their patent-protected competitive allele specific PCR system (KASPar) (http://www.kbioscience.co.uk/index.html).

### Statistics

Data on smoking habits and lung function collected during the surveys from 1965 to 1990 have been used for analyses. To study the changes in smoking habits such as quitting smoking within smokers and restarting smoking within ex-smokers, we compared the information of two successive surveys. The paired observations had a minimum interval of 3 years and every subject who had smoked at any time contributed to the analyses with a maximum of 7 paired observations. Analyses of the paired observations have been performed separately for smokers and ex-smokers: To investigate quitting smoking we selected all paired observations in which the subject smoked at the first observation of 2 successive surveys. The smoking habit (i.e. smoking or ex-smoking) at the nearest follow-up survey (i.e. the second observation of two successive surveys) was used as dependent variable in our analyses. To investigate restarting smoking we selected all paired observations in which the subject was an ex-smoker at the first of 2 successive surveys. Again, the smoking habit (i.e. ex-smoking or restarted smoking) at the nearest follow-up survey was the dependent variable in our analyses.

### 1. SNPs in the *nAChR* cluster and changes in smoking habits in smokers and ex-smokers

Generalized estimating equations (GEE) were used to investigate in smokers and ex-smokers separately the associations of the SNPs in the *nAChR* cluster with quitting and restarting smoking, respectively. This method takes into account the dependence of multiple measurements within one subject and adjusts for the fact the intervals between the observations are not constant and the number per subject was variable. In the GEE model, an OR>1 should be interpreted as an increased chance to stop or restart smoking and an OR<1 should be interpreted as a decreased chance to stop or restart smoking. In the GEE model, the smoking habit was the dependent variable (categorical). We adjusted our analyses for gender and the time between two successive surveys.

### 2. SNPs in the *nAChR* cluster and the annual FEV_1_ decline in smokers and ex-smokers

Linear mixed-effects models (LME) were used in smokers and ex-smokers separately to investigate the associations of the SNPs in the *nAChR* cluster with the annual FEV_1_ decline (defined as the difference in FEV_1_ between the 2^nd^ and 1^st^ observation of two successive surveys divided by the time in years between these surveys). In the LME model, the annual FEV_1_ decline was the dependent variable (continuous). We adjusted our analyses for gender, the time between two successive surveys and age and height at the first of two successive surveys. Quitting and restarting smoking were also used in the analyses as independent variables for smokers respectively ex-smokers. In the LME model, a negative regression coefficient should be interpreted as an excess FEV_1_ decline per year and a positive regression coefficient as less FEV_1_ decline per year as compared to the wildtype. In the LME model effect estimates are considered significant if the confidence interval does not include 0. Since E, estimated from the LME model, can be interpreted as a regression coefficient, we indicated it as a B throughout the paper to improve consistency of the presented results and ease of interpretation.

### 3. Changes in smoking habits and the annual FEV_1_ decline in smokers and ex-smokers

LME models were used to investigate the associations of quitting and restarting smoking with the annual FEV_1_ decline separately in smokers and ex-smokers. The analyses were adjusted for gender, age and height at the first of two successive surveys and the time between two successive surveys.

### 4. SNPs in the *nAChR* cluster and cigarettes smoked per day, packyears and COPD at the last survey

We used linear regression to assess the associations of the SNPs and number of cigarettes smoked per day at the last survey in current smokers. The associations of the SNPs with packyears were performed in ever smokers (current smokers and ex-smokers together). We adjusted for age and gender. We used chi-square tests to assess the differences in the prevalence of the SNPs between subjects with (FEV_1_/FVC<70%) and without (FEV_1_/FVC>70%) COPD. In the linear regression model, a negative regression coefficient should be interpreted as a lower number of cigarettes per day and less packyears, and a positive regression coefficient should be interpreted as a higher number of cigarettes per day and higher number of packyears.

To asses the associations of the SNPs in the *nAChR* cluster with changes in smoking habits, with the annual FEV_1_ decline in smokers and ex-smokers, and with the daily number of cigarettes and packyears at the last survey we used the general genetic model where heterozygote and homozygote variants were coded separately as dummy variables and compared to the homozygote wild type.

All analyses were performed using SPSS version 16.0 for Windows and values of p<0.05 (tested 2-sided) were considered statistically significant. Linkage Disequilibrium (LD) plots (a threshold of 0.8 for the correlation coefficient (r^2^) and Hardy Weinberg Equilibrium (HWE) tests were performed with Haploview (version 4.2) [33].

LME models were used with a random intercept at the subject level, assuming data are missing at random. We included only FEV_1_ measurements from the age of 30 years onwards because it is assumed that the maximal lung function is reached before that age and the FEV_1_ is considered to be either in the plateau or decline phase [34].

## Supporting Information

Figure S1 **Linkage disequilibrium plot and correlation coefficients (r^2^) for 3 polymorphisms in the** ***nAChR*** **cluster genotyped in Vlagtwedde-Vlaardingen cohort (n = 1,390).** The location of the single nucleotide polymorphisms is given for the HapMap Data Release February 2009.
(TIF)

Table S1 **Prevalence of the** ***nAChR*** **SNPs in Vlagtwedde – Vlaardingen.** N = number of subjects.
(DOCX)

Table S2 **The effect of the** ***nAChR*** **SNPs on smoking habits in smokers (pairs = 3393) and ex-smokers (pairs = 1468).** GEE model, adjusted for gender, time between 2 consecutive available visits; a = heterozygote vs. wild-type; b = homozygote variant vs. wild-type.
(DOCX)

Table S3 **Annual FEV_1_** **declines in smokers and ex-smokers.** Nr FEV_1_ declines refers to the total number of annual FEV_1_ declines available; Nr. subjects refers to smokers or ex-smokers who have at least 1 annual FEV_1_ decline available.
(DOCX)

Table S4 **Differences in annual FEV_1_ decline according to smoking status.** B = regression coefficient; LME model adjusted for gender, height and age at the first of two successive surveys and time between two successive surveys. The results showing a significant association are depicted in bold.
(DOCX)
