# Supplementary figures and images for: Nicotinic Acetylcholine Receptor Variants Are Related to Smoking Habits, but Not Directly to COPD
Source: PLoS One. 2012 Mar 15;7(3):e33386. doi: 10.1371/journal.pone.0033386 (PMC3305325; doi:10.1371/journal.pone.0033386)

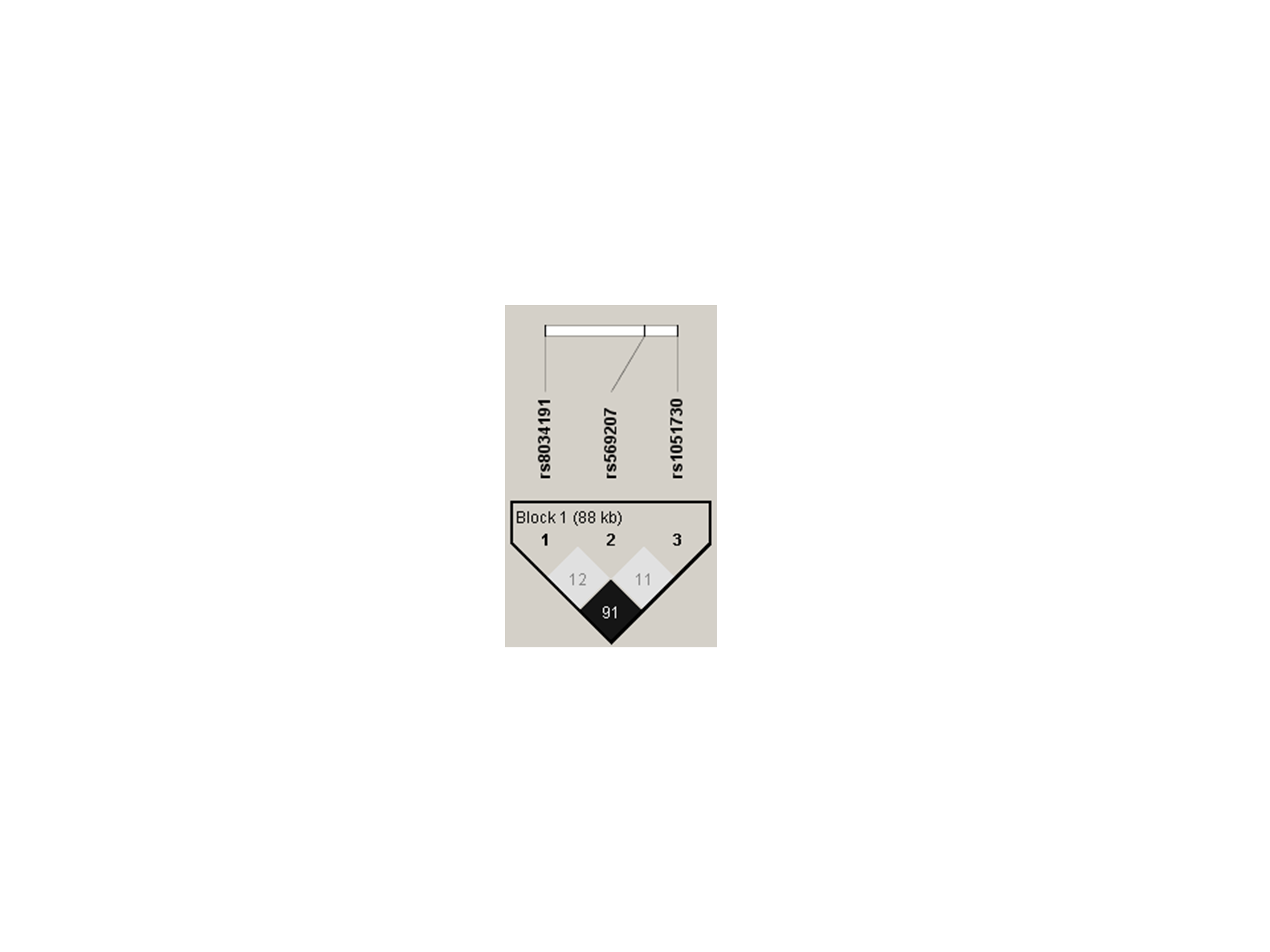

Supplement: Figure S1 — Linkage disequilibrium plot and correlation coefficients (r2) for 3 polymorphisms in the nAChR cluster genotyped in Vlagtwedde-Vlaardingen cohort (n = 1,390). The location of the single nucleotide polymorphisms is given for the HapMap Data Release February 2009. (TIF) [file pone.0033386.s001.tif]
