# Supplementary material for: Nicotinic Acetylcholine Receptor Variants Are Related to Smoking Habits, but Not Directly to COPD
Source: PLoS One. 2012 Mar 15;7(3):e33386. doi: 10.1371/journal.pone.0033386 (PMC3305325; doi:10.1371/journal.pone.0033386)
Supplement: Table S1 — Prevalence of the nAChR SNPs in Vlagtwedde – Vlaardingen. N = number of subjects. (DOCX) [file pone.0033386.s002.docx]

**Table S1: Prevalence of the *nAChR* SNPs in Vlagtwedde – Vlaardingen**

| SNPs | Gene | Genotypes | N=1390 (%) |
| --- | --- | --- | --- |
| rs569207 | CHRNA5 | TT | 735 (56.8) |
|  |  | TC | 487 (37.6) |
|  |  | CC | 72 (5.6) |
| rs1051730 | CHRNA3 | CC | 685 (51.5) |
|  |  | CT | 537 (40.4) |
|  |  | TT | 108 (8.1) |
| rs8034191 | LOC123688 | GG | 666 (50.3) |
|  |  | GA | 540 (40.8) |
|  |  | AA | 117 (8.9) |
