# Supplementary material for: Nicotinic Acetylcholine Receptor Variants Are Related to Smoking Habits, but Not Directly to COPD
Source: PLoS One. 2012 Mar 15;7(3):e33386. doi: 10.1371/journal.pone.0033386 (PMC3305325; doi:10.1371/journal.pone.0033386)
Supplement: Table S2 — The effect of the nAChR SNPs on smoking habits in smokers (pairs = 3393) and ex-smokers (pairs = 1468). GEE model, adjusted for gender, time between 2 consecutive available visits; a = heterozygote vs. wild-type; b = homozygote variant vs. wild-type. (DOCX) [file pone.0033386.s003.docx]

**Table S2: The effect of the *nAChR* SNPs on smoking habits in smokers (pairs=3393) and ex-smokers (pairs=1468)**

| SNPs | Model | **quit smoking** | | | **restart smoking** | | |
| --- | --- | --- | --- | --- | --- | --- | --- |
|  |  | OR | 95%CI | p-value | OR | 95%CI | p-value |
| **rs569207** | a | 0.97 | 0.76 - 1.22 | 0.782 | 1.40 | 0.93 – 2.09 | 0.105 |
|  | b | 1.58 | 1.05 – 2.38 | **0.027** | 0.58 | 0.25 – 1.33 | 0.200 |
| **rs1051730** | a | 0.93 | 0.73 – 1.61 | 0.520 | 0.99 | 0.65 – 1.48 | 0.960 |
|  | b | 0.64 | 0.42 – 0.97 | **0.039** | 0.77 | 0.34 – 1.73 | 0.529 |
| **rs8034191** | a | 1.004 | 0.79 – 1.27 | 0.972 | 1.12 | 0.74 – 1.69 | 0.572 |
|  | b | 1.180 | 0.81 - 1.75 | 0.375 | 0.88 | 0.42 – 1.82 | 0.740 |
