# Supplementary material for: Nicotinic Acetylcholine Receptor Variants Are Related to Smoking Habits, but Not Directly to COPD
Source: PLoS One. 2012 Mar 15;7(3):e33386. doi: 10.1371/journal.pone.0033386 (PMC3305325; doi:10.1371/journal.pone.0033386)
Supplement: Table S3 — Annual FEV1 declines in smokers and ex-smokers. Nr FEV1 declines refers to the total number of annual FEV1 declines available; Nr. subjects refers to smokers or ex-smokers who have at least 1 annual FEV1 decline available. (DOCX) [file pone.0033386.s004.docx]

**Table S3: Annual FEV_1_ declines in smokers and ex-smokers**

| SNP | Genotype | 786 smokers with 3276 annual FEV_1_ declines | | 467 ex-smokers with 1426 annual FEV_1_ declines | |
| --- | --- | --- | --- | --- | --- |
|  |  | Nr. subjects | Nr. FEV_1_ declines | Nr. subjects | Nr. FEV_1_ declines |
| rs569207 | TT | 418 | 1767 | 242 | 743 |
|  | TC | 275 | 1171 | 155 | 455 |
|  | CC | 39 | 140 | 32 | 114 |
| rs1051730 | CC | 390 | 1586 | 232 | 709 |
|  | CT | 312 | 1316 | 183 | 545 |
|  | TT | 60 | 286 | 33 | 101 |
| rs8034191 | GG | 375 | 1521 | 225 | 707 |
|  | GA | 309 | 1298 | 183 | 541 |
|  | AA | 65 | 296 | 40 | 121 |
