# Supplementary material for: Nicotinic Acetylcholine Receptor Variants Are Related to Smoking Habits, but Not Directly to COPD
Source: PLoS One. 2012 Mar 15;7(3):e33386. doi: 10.1371/journal.pone.0033386 (PMC3305325; doi:10.1371/journal.pone.0033386)
Supplement: Table S4 — Differences in annual FEV1 decline according to smoking status. B = regression coefficient; LME model adjusted for gender, height and age at the first of two successive surveys and time between two successive surveys. The results showing a significant association are depicted in bold. (DOCX) [file pone.0033386.s005.docx]

Table S4: Differences in annual FEV1 decline according to smoking status

| **Differences in annual FEV_1_ decline** | | | |
| --- | --- | --- | --- |
|  | B | 95%CI | p-value |
| Smokers vs. Never smokers | -6.4 | -12.5 – -0.3 | **0.039** |
| Ex-smokers vs. Never smokers | 1.5 | -5.3 – 8.4 | 0.662 |
| Smokers vs. Ex-smokers | -7.9 | -13.8 – -2.0 | **0.009** |
